# Molecular profiling and personalized medicine within EURACAN in the SPECTA Arcagen study in comparison to the France Genomic Medicine 2025 Plan in the AURAGEN platform in peritoneal mesotheliomas

**DOI:** 10.1016/j.tranon.2026.102825

**Published:** 2026-05-18

**Authors:** Seddig Momenkhan, Marie Morfouace, Vahan Kepenekian, Olivier Glehen, Laurent Villeneuve, Julio Oliveira, Sabine Tejpar, Sylvie Isaac, Juliette Fontaine, Jean-Yves Blay, Julien Péron, Nazim Benzerdjeb

**Affiliations:** a Department of Pathology, Institut of Pathologie Multisite, Hospices Civils de Lyon, Hôpital Lyon Sud, Pierre-Bénite, France; b European Organisation for Research and Treatment of Cancer, Brussels, Belgium; c Gustave Roussy, Villejuif, France; d CICLY - EA3738, Université Claude Bernard Lyon I (UCBL1), Lyon, France; e Department of Digestive Surgery, CNR RENAPE, Hôpital Lyon Sud, Pierre-Bénite, France; f Epidemiology and Clinical Research Department, Public Health Department, Hospices Civils de Lyon, Lyon, France; g Instituto Português de Oncologia, Department of Medicine, Porto, Portugal; h KU Leuven, Department of Oncology, Leuven, Belgium; i Centre Léon Bérard & Université Claude Bernard Lyon I (UCBL1), Lyon, France; j Department of Medical Oncology and Research Center on Healthcare Performance, (RESHAPE) INSERM U1290, Hospices Civils de Lyon, Pierre-Bénite, France

**Keywords:** Molecular alteration, Mesothelioma, Personalized medicine

## Abstract

Peritoneal mesothelioma (PM) is a rare aggressive cancer with limited therapeutic options upon progression. Two genomic profiling initiatives were launched to implement personalized medicine in rare cancers: the EURACAN molecular profiling and personalized medicine pathway and the France Genomic Medicine 2025 plan (AURAGEN). The aim of this study was to describe initial findings in PM and to compare these approaches in a real-world implementation setting. Herein, we present the results from a cohort of 56 patients with PM. Females represented 55.4% of patients; mean age was 55.9 years. The European (Arcagen) study profiled 26 patients and the AURAGEN platform profiled 42 patients. Genomic sequencing was performed using Foundation Medicine for the Arcagen cohort and whole-genome sequencing (WGS) for the AURAGEN cohort. Arcagen used FFPE tissue or blood depending on material availability, whereas AURAGEN relied on fresh frozen tumor tissue with paired blood (and RNA sequencing when feasible). Both cohorts displayed a predominance of alterations in BAP1, NF2, and CDKN2A/B. One patient with an ALK::STRN fusion was treated with Alectinib. AURAGEN identified a larger number of altered genes overall, while the proportion of therapeutic targets was similar between approaches. In conclusion, comprehensive molecular profiling offers potential therapeutic strategies for PM patients. Panel-based profiling and pan-genomic WGS appear complementary in real-world practice, each with distinct strengths and limitations.

## Introduction

Peritoneal mesothelioma (PM) is a rare and aggressive malignancy affecting the peritoneum [1] It comprises about 10–15% of all mesotheliomas, thereby being the second most common site of origin after the pleura [2] Treatment options have evolved, and a locoregional approach combining complete cytoreductive surgery (CRS) with hyperthermic intraperitoneal chemotherapy (HIPEC) was reported to improve survival rates up to 50 months [3] Immune checkpoint inhibitors have shown some efficacy in this disease, and represent a new therapeutic option in addition to platinum and pemetrexed chemotherapy [4,5] However, when the disease is not amenable to CRS, the chances of long-term disease control remain modest.

Rare cancers such as PM are often underreported in genomic profiling studies, contributing to the scarcity of the available targeted therapies. The use of personalized approaches through assessment of the molecular profile of tumors shows potential promises. However, information on the molecular profile of PM has historically been scarce; recent studies described it, providing valuable data that could help the identification of new therapeutic targets for PM [[6], [7], [8], [9]] Nonetheless, no molecular study has been conducted with the intention to treat.

The aim of the present study was to identify new therapeutic targets for patients with PM with an intention to treat. In this context, several approaches can be proposed, including that of the Foundation Medicine, in which the analysis is performed on a large panel of genes. This approach is used by Arcagen, a European Organisation for Research and Treatment of Cancer (EORTC) SPECTA prospective multicohort and multicenter study that aims to address this gap by conducting genomic profiling and using a molecular tumor board (MTB) to guide treatment recommendations for patients with rare cancers in Europe. In addition, AURAGEN, a French organization for cancer treatment, focuses on genomic profiling using a pan-genomic analysis and an MTB for patients with rare cancers in France. Herein, we present the initial findings of these two approaches for the rare PM cohort. Because Arcagen and AURAGEN use different technologies and reporting frameworks (targeted panel-based profiling versus WGS-based pan-genomic analysis), comparisons are presented descriptively to highlight real-world implementation strengths and limitations rather than to infer analytically harmonized differences.

## Materials and methods

### Patients selection

According to the feasibility and availability criteria of Arcagen and AURAGEN, we retrospectively included patients diagnosed with, relapsing from, treated for, or followed for peritoneal mesothelioma (PM). Diagnoses were assessed and confirmed by expert pathologists. Tumor material was reviewed by expert pathologists to assess feasibility for comprehensive genomic profiling (notably tumor cellularity and sample adequacy). Ethical approval was obtained from the institutional review board; patients provided written informed consent and were informed of the use of their data (23-5368).

#### Arcagen cohort

Tissue sampling for the Arcagen cohort was conducted through five affiliated centers of the EURACAN network: Hospices Civils de Lyon (HCL, Lyon, France), Centre Léon Bérard (Lyon, France), Institut Bergonié (Bordeaux, France), U.Z. Leuven (Leuven, Belgium), and Instituto Português de Oncologia do Porto Francisco Gentil (Porto, Portugal), within the EORTC–SPECTA platform. The Arcagen study was performed between June 2019 and March 2022. The aim was to assess the frequency of genomic alterations, high tumor mutational burden (TMB), and microsatellite instability (MSI), as well as potential actionable mutations in patients diagnosed with advanced rare PM [10] Analysis was performed on tumor formalin-fixed paraffin-embedded (FFPE) tissue samples (when available and obtained within 2 years, n = 17) or blood samples (n = 4), using the Foundation Medicine platform (F1CDx for tissue; F1LCDx for blood). FFPE material was obtained either from primary or recurrent tumor. A hematoxylin and eosin (H&E) stained section was prepared for each FFPE block and reviewed by an expert pathologist to assess tissue quality and tumor content. Based on the pathologist review, high-quality samples, with a minimum surface area of 25 mm² and containing at least 20% of viable tumor cells were selected for molecular analysis [10] If the block was older than 2 years or of poor-quality, a blood sample was taken for genomic analysis. FoundationOne CDx is performed on DNA extracted from FFPE tumor tissue with a reported DNA input requirement of 50–1000 ng, and sequencing targets >500 × median coverage with >99% of exons sequenced at >100 × . For blood-based profiling (FoundationOne Liquid CDx), analysis is performed on circulating tumor DNA from plasma, and performance depends on tumor DNA fraction and pre-analytical handling; therefore, certain biomarkers may be non-reportable in low-shedding settings. For Arcagen, alterations and clinical annotations were extracted from the final Foundation Medicine clinical reports (F1CDx for tissue; F1LCDx for blood). Variants reported as variants of uncertain significance (VUS), when listed separately by the provider, were not considered clinically relevant and were excluded from cross-cohort comparisons and actionability summaries.

#### AURAGEN cohort

The France Genomic Medicine 2025 Plan (PFMG2025) is a national policy organizing and financing the access to whole genome sequencing (WGS) in care settings [11] Two genome platforms, the Sequencing, Omics, Information Analysis (SeqOIA) and the *Auvergne Rhône-Alpes Génomique* (AURAGEN), have been created to cover different geographic areas in France. These platforms enable high-throughput genomic sequencing within predefined clinical indications in the PFMG2025 framework.Forty-two PM patients were enrolled in AURAGEN; sequencing was successful in 35 cases. Analysis was conducted on fresh frozen tumor tissue samples and blood samples using the AURAGEN platform, which uses the NovaSeq™ 6000 Sequencing System (Illumina, Inc., San Diego, CA, US) for WGS analysis, in addition to examining gene signatures such as TMB and MSI. WGS was performed for all included cases, and RNA sequencing was performed when feasible. Tissue samplings were collected from patients with PM at HCL. A rapid staining was performed on the frozen specimen by an expert pathologist to assess tissue quality and tumor content. Based on the pathologist review, high quality samples containing at least 10% of viable tumor cells were selected for molecular analysis. Relevant clinical data, containing details on the received therapies, follow-up records, and consultation history, was retrieved from the computerized medical records of the patients. Bioinformatics processing was performed through platform-standardized automated pipelines, followed by specialist medical interpretation and clinical reporting according to the PFMG2025 framework. For comparative analyses in this manuscript, we restricted results to clinically relevant alterations retained in the final report and excluded variants of uncertain significance (VUS) from gene counts and cross-cohort summaries [12]

#### Statistical analysis

Data were analyzed using univariate methods. Categorical variables were compared between groups using Fisher's exact test, and continuous variables were compared using the Student's t-test or Mann-Whitney U test, as appropriate. Effect sizes were calculated and reported as odds ratios (OR) with 95% confidence intervals (CI) for categorical variables and Cohen's d for continuous variables to assess the clinical magnitude of observed differences. Given the exploratory and descriptive nature of this comparative analysis, no adjustment for multiple comparisons was applied. This approach was chosen as the analysis aimed to characterize the two cohorts rather than test specific a priori hypotheses. A detectable effect size (post-hoc) analysis was performed to assess the study’s ability to detect between-group differences. With the available sample size (n = 56; Arcagen n = 21, AURAGEN n = 35) and α=0.05, the study had 80% power to detect a medium effect size (Cohen's d ≥ 0.77 for continuous variables; OR ≥ 4.0 or ≤ 0.25 for categorical variables with balanced proportions). The limited sample size represents a constraint on detecting smaller, yet potentially clinically relevant, differences between groups. All statistical analyses were performed using R statistical software (version 4.2.0) with the RcmdrPlugin.EZR package [13]. A two-sided *p-value* of <0.05 was considered statistically significant.

## Results

### Clinical profile of the cohort

Fifty-six patients were enrolled for comprehensive genomic profiling. Sequencing failed for 5 Arcagen cases (5/26, 19.2%) and 7 AURAGEN cases (7/42, 16.7%). Sequencing failure was defined as the inability to generate a reportable molecular profile. In addition, specific biomarkers (e.g., MSI/TMB) could be reported as ‘cannot be determined’ when confidence thresholds were not met despite otherwise successful sequencing. The most common reasons for failure were low tumor cellularity and/or insufficient nucleic-acid quality/quantity; degraded FFPE material was a contributing factor in tissue-based Arcagen cases. Hence, 21 patients were evaluable by the Arcagen platform and 35 patients by the AURAGEN platform. The mean age of patients with PM was 55.9 years (range 24–80), it was similar in both cohorts. There was a significant difference regarding the sex ratio between the Arcagen and the AURAGEN cohort (p = 0.021). The disease extension, expressed through the mean Peritoneal Cancer Index (PCI), was significantly lower for the AURAGEN cohort (18.6) compared to the Arcagen (22.8; p < 0.05). There was no difference in terms of surgical treatment between the cohorts. Most patients in the Arcagen cohort received neo-adjuvant chemotherapy (18/21, 85.7%) and only one patient was treated using a targeted therapy, Alectinib, for an *ALK* fusion. In addition, one patient of the AURAGEN cohort received a combination of Nivolumab and Ipilimumab (Table 1). To provide a descriptive overview of clinical trajectories, we summarized key treatment steps and follow-up over time using swimmer plots for the Arcagen and AURAGEN cohorts (Fig. 2). This visualization is intended to be descriptive and does not constitute a comparative survival analysis.

**Table 1:** Clinical features of Arcagen and AURAGEN cohorts.

	All n = 56	Arcagen n = 21	AURAGEN n = 35	*P-value*	OR (95% CI) / Effect Size d
Sex, n (%) Male Female	25 (44.6%) 31 (55.4%)	14 (66.7%) 7 (33.3%)	11 (31.4%) 24 (68.6%)	0.021	4.36 (1.40–13.58)
Mean age (range)	55.9 (24–80)	55 (24–78)	56.8 (21–80)	0.576	d = −0.15
Histological subtype, n (%) Epithelioid Biphasic Sarcomatoid Unspecified Multicystic	41 (73.2%) 6 (10.7%) 0 1 (1.8%) 8 (14.3%)	18 (85.9%) 1 (4.7%) 0 1 (4.7%) 1 (4.7%)	23 (65.7%) 5 (14.3%) 0 0 7 (20.0%)	0.131	3.13 (0.82–11.93)
Mean PCI (range) NA	20.7 (2–39) 6	22.8 (11–39) 5	18.6 (2–39) 1	0.118	d = 0.43
Surgery, n (%) Cytoreduction HIPEC	55 (98.2%) 47 (83.9%)	20 (99.2%) 13 (61.9%)	35 (100%) 34 (97.1%)	0.644	0.05 (0.01–0.43)
Neo-adjuvant Chemotherapy Permetrexed Platin	35 (62.5%) 35 (62.5%)	18 (85.7%) 18 (85.7%)	17 (48.5%) 17 (48.5%)	0.571	6.35 (1.71–23.60)
Targeted therapy Immunotherapies	1 (1.8%) 1 (1.8%)	1* (4.7%) 0	0 1** (2.8%)		- -

CI = Confidence Interval; d = Cohen's d effect size; OR = Odds Ratio; PCI, peritoneal cancer index; NA, Not available; HIPEC, Hyperthermic Intraperitoneal Chemotherapy; *Alectinib for *ALK* fusion; **Nivolumab and Ipilimumab. OR >1 favors Arcagen group; OR <1 favors AURAGEN group.

### Molecular profile of the cohorts

**Fig. 1:**
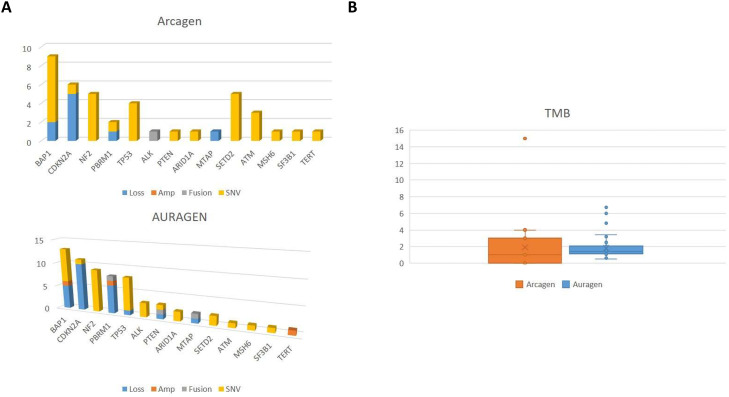
Molecular profiling of the cohorts: (A) Frequency of alterations per cohort, Arcagen (top panel) and AURAGEN (bottom panel). (B) TMB in both cohorts. Legend: Amp, Amplification; Loss; Gene loss; SNV, single nucleotide variant; and TMB, Tumor mutation burden.

**Fig. 2:**
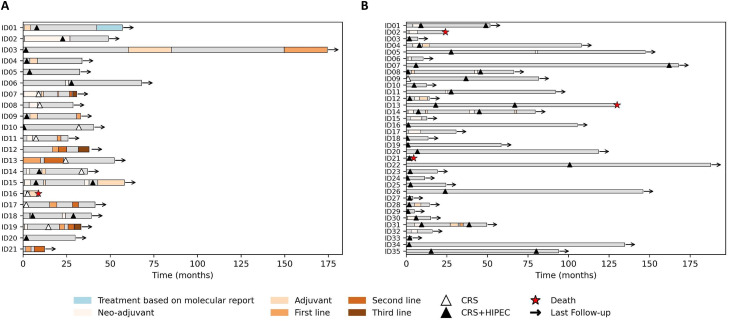
Timelines peritoneal mesothelioma stratified by cohort: (A) Arcagen cohort, and (B) AURAGEN cohort. Legend: CRS, Cytoreductive surgery; and CRS+HIPEC, Cytoreductive surgery and hyperthermic intraperitoneal chemotherapy.

For cross-cohort comparisons and gene counts, variants reported as VUS were excluded, and analyses were restricted to clinically relevant alterations retained in the final clinical reports. A mutational status with at least one clinically relevant mutation was identified in the majority of patients from both cohorts (>77%). Given the small number of blood-based cases (n = 4) and the descriptive nature of this analysis, Arcagen FFPE- and blood-based results are presented together. The TMB status and MSI were obtained in approximately 75% of all cases (Fig. 1A). The TMB status was low, except for one patient from the Arcagen cohort (Fig. 1B, Table 2). The MSI status was stable, except for one patient from the AURAGEN cohort (Table 2). Consistent with its broader genomic scope, the AURAGEN platform identified a larger number of genes harboring reported alterations than Arcagen (100 and 25, respectively, Fig. 1A). However, no significant difference was found regarding the number of targetable alterations between both cohorts. The median turn-around time (TAT) from patient enrollment in the study to delivery of the molecular report was 12 days for Arcagen (range 8–48), and 50 days for AURAGEN (range 27–68). Importantly, TAT reflects an end-to-end clinical workflow (from sample availability/logistics to final report sign-out) and therefore does not represent sequencing instrument runtime alone. This shorter end-to-end TAT in Arcagen allowed for molecular tumor board discussion and therapeutic proposals in case of progression. One case from the Arcagen cohort harbored the *ALK*::*STRN* fusion. Treatment was switched to Alectinib, and the patient remained on treatment 15 months later at the time of data cutoff (Fig. 1A, blue bar). Four patients from the AURAGEN cohort were harboring gene fusions (*MTAP*::*CDKN2B-AS1, PBRM1*::*POC1A, PTEN*::*RNLS*, and *EWSR1*::*YY1*), but no molecularly targeted therapy was initiated based on these fusions. Swimmer plots for all patients in the study are shown in Fig. 2. The most frequently detected mutations were found in *BAP1* (n = 9, 42.8% of patients), *CDKN2A/B* (n = 6, 28.6%), *NF2* (n = 5, 23.8%), and *SETD2* (n = 5, 23.8%) in the Arcagen cohort and in *BAP1* (n = 13, 37.1%), *CDKN2A* (n = 11, 31.4%), *TP53* (n = 8, 22.8%), and *NF2* (n = 9, 25.7%) in the AURAGEN cohort (Fig. 2A). The potential treatments according to the molecular profile of the two cohorts are summarized in Table 3, with 44 patients presenting targetable alterations either for clinical trials (n = 22), off-label (n = 21) or approved therapy (n = 1).

**Table 2:** Molecular alterations status of Arcagen and AURAGEN cohorts.

	All n = 56	Arcagen n = 21	AURAGEN n = 35	*P-value*	OR (95% CI) / Effect Size d
Mutational status With clinically relevant mutation Without clinically relevant mutation	46 (82%) 10 (18%)	19 (90.5%) 2 (9.5%)	27 (77%) 8 (23%)	0.207	2.82 (0.54–14.78)
TMB status High (15 Mut/MB) Low (0–6 Mut/MB) Failed	1 (1.8%) 42 (75.0%) 13 (23.2%)	1 (4.7%) 14 (66.8%) 6 (28.5%)	0 28 (80%) 7 (20%)	0.302	0.50 (0.16–1.58)
MSI status Stable Unstable Failed	42 (75.0%) 1 (1.8%) 13 (23.2%)	15 (71.4%) 0 (0%) 6 (28.6%)	27 (77.14%) 1 (2.85%) 7 (20%)	0.584	0.74 (0.23–2.39)

CI = Confidence Interval; d = Cohen's d effect size; OR = Odds Ratio; TMB, Tumor mutational burden; MSI, Microsatellite instability; Mut/MB, mutations per megabase. OR >1 favors Arcagen group; OR <1 favors AURAGEN group.

**Table 3:** Treatments according to the molecular profile.

	All n = 53	Arcagen n = 21	AURAGEN n = 35
Treatment in EU in tumor type	**1** *ALK*::*STRN* fusion	**1** *ALK*::*STRN* fusion	**0**
Treatment in EU in other tumor types	**21** TMB, 1 MSI-H, 1 *BRCA*, 1 *NF1*, 3 *ATM*, 2 *FBXW7*, 5 *NF2*	**8** *ATM*, 1 *BRCA*, *NF2*, 1 *NF1*, TMB	**13** 1 *ATM*, 1 *EZH2*, 2 *FBXW7*, 9 *NF2*, 1 MSI-H
Clinical trial	**22** 3 *ARID1A*, 19 *BAP1*	**7** 6 *BAP1*, 1 *ARID1A*	**15** 2 *ARID1A*, 13 *BAP1*
No treatment proposed	**3**	**3**	**0**
No clinically relevant mutations	**10**	**2**	**7**

TMB, Tumor mutational burden; MSI, Microsatellite instability; EU, European Union. Numbers in parentheses indicate the count of patients with each specific molecular alteration.

## Discussion

Because these two approaches are not analytically harmonized (different assay scope, depth, and reporting), cross-cohort contrasts should be interpreted as real-world comparisons of reported clinically relevant alterations rather than as platform-equivalent frequency estimates. In this context, screening for genomic alterations in PM through two different methods identified a similar frequency of therapeutic targets. The two cohorts being the Arcagen study that used the Foundation Medicine approach based on FFPE or blood analysis (large NGS panel), and the AURAGEN (WGS) study using a pan-genomic analysis based on fresh sampling. However, the AURAGEN cohort identified a greater number of genes not related to a specific treatment, which provides clinicians with new molecular alterations that may lead to the identification of novel therapeutic targets.

The goal of both Arcagen and AURAGEN studies was to provide therapeutic options for patients and therefore, TAT between patient enrolment and results of the molecular profiling is crucial. These TATs reflect end-to-end real-world workflows (including sample availability, logistics, laboratory processing, bioinformatics, interpretation, and report sign-out) rather than intrinsic sequencing technology performance alone. The TAT was shorter for Arcagen at 12 days (range 8–48), while it was longer for AURAGEN at 50 days (range 27–68), allowing for possible treatment adaptation, particularly for patients in need of new therapies due to the failure of standard-of-care treatment in two phases: rapidly for classic targets, and subsequently for less conventional targets. This approach makes the most of the advantages of each method: the speed of Foundation Medicine, and the comprehensiveness of AURAGEN platform.

As patients who might benefit the most from advanced molecular profiling are patients with advanced diseases, re-biopsying these patients can often be challenging. Therefore, the Arcagen study decided to use either a recent archival FFPE block (<2 years old) or a blood sample to perform a large scale NGS. In the AURAGEN cohort, the workflow relied on fresh frozen tumor tissue with paired blood, which was the standard pathway in the present PM series.

One *ALK* fusion was identified in our study (1.7%), and was also detected in similar rate (1.13%) in another large cohort of PM patients [6] Despite the low prevalence of this alteration in PM, the *ALK* fusion case illustrates the feasibility of molecularly guided treatment selection in this real-world setting (patient remained on alectinib 15 months after treatment switch at data cutoff; Fig. 2A, blue bar), consistent with prior reports [[14], [15], [16], [17]] The present study also observed a higher occurrence of mutations in *BAP1* (39%), *CDKN2A* (30%), and *NF2* (25%) as documented in other studies [[18], [19], [20]]. Recent findings in patients with low-risk mesothelioma have additionally highlighted a high prevalence of *CDKN2A/B* deletions, notably in pleural mesothelioma [1,9,20]

For most of those frequent alterations, therapies are not yet approved. In the case of *BAP1* mutation, while no direct inhibitors exist, therapies targeting molecular pathways influenced by *BAP1* are available. This gene is linked to *BRCA1*-mediated pathways and plays a crucial role in homologous recombination repair (HRR), a key mechanism in DNA repair [21,22] Thus, tumors deficient in both *BAP1* and *BRCA1* may respond to *PARP* inhibitors, with encouraging outcomes observed in a phase 2 clinical trial (TOPARP-B) [30], An ongoing trial (TALAMESO), is currently evaluating talazoparib following first line platinum based chemotherapy in malignant mesothelioma (NCT04462809). Nonetheless, *in-vitro* studies show variable results regarding the effectiveness of PARP inhibitors according to the *BAP1* status [23,24] EZH2, often overexpressed in *BAP1*-deficient tumors, represents another viable target. Preclinical studies indicate that *BAP1-*deficient mice have improved sensitivity to EZH2 inhibitors [25] In a phase 2 trial, 74 patients with *BAP1*-deficient mesothelioma were treated by the EZH2 inhibitor tazemetostat as a single-agent therapy, achieving a disease control rate of 51% at 12 weeks and 25% at 24 weeks [26] However, only two partial responses were reported, and no complete response, suggesting that these moderate outcomes are not directly tied to *BAP1* deficiencies, and biomarkers for predicting responses to tazemetostat remain to be identified. Additionally, the role of *BAP1* in HRR has prompted its study as a potential biomarker for chemotherapy response; wild-type *BAP1* is linked to increased sensitivity to gemcitabine in mesothelioma cell lines, though this finding has yet to be confirmed in patients with PM [27,28] For *NF2* mutation, some clinical and preclinical research indicates that NF2 inactivation may correlate with a response to mTOR inhibitors [29,30] Everolimus and temsirolimus, both mTOR inhibitors, have received FDA approval for the treatment of various cancers including neuroendocrine tumors of the gastrointestinal tract or lung, *HER2*/neu-negative breast cancer, and renal cell carcinoma. Nonetheless, a phase 2 trial involving patients with pleural mesothelioma reported only a 2% response rate to everolimus [31] No activating mutations in oncogenes such as *EGFR* and *BRAF* was detected in the present study, although found in 1–2% of patients in other studies [6] Notably, it is difficult to guarantee the therapeutic effectiveness of drugs targeting these mutations; as for *EGFR* mutations, either there is a low response rate, making it less effective than standard chemotherapy, or there is a stable response [[32], [33], [34]]

In AURAGEN, we also observed several non-classical alterations such as *EWSR1*::*ATF1* fusion. This alteration has been reported in some sarcomas such as angiomatoid fibrous histiocytoma, and treatment recommendation with interleukin-6 (IL-6) receptor antibody tocilizumab could be proposed [[35], [36], [37], [38]] Moreover, our group previously reported good results when using tocilizumab for one patient with *EWSR1*-rearranged epithelial mesothelioma [39], which suggests that tocilizumab may be effective for the patient with the *EWSR1* fusion. To our knowledge, no clinical trial had, to date, investigated the specific utility of anti-IL-6 therapy in PM, highlighting the need for more clinical research is this rare disease.

Despite several limitations, the main strength of the present study is the in-depth analysis of PM molecular profile using two different platforms, one at the European level, and the other one at a national level, conducted with the intention to treat in current daily practice. It provided a more comprehensive dataset compared with recently published studies reporting larger cohorts, which can be valuable for the guidance of future therapeutic developments [20,40]. One notable limitation is the use of three different sample types, which may limit comparisons. This heterogeneity may contribute to failures and/or non-reportability of specific signatures and therefore represents a potential source of bias when interpreting cross-cohort differences. In addition, the between-cohort statistical comparisons were exploratory and were not adjusted for multiple testing; p-values should therefore be interpreted cautiously, with emphasis placed on the reported effect sizes. Clinical outcomes (response and survival) were not analyzed statistically because of limited sample size, heterogeneous treatments and timing of profiling; outcome information is therefore presented descriptively, and prospective studies are needed to assess clinical outcomes and the impact of profiling-guided strategies. However, when focusing on clinically relevant alterations and therapeutic targets, we did not observe a clear difference in the proportion of targetable alterations reported between approaches. The study also raises the question of the best sample to use for routine genomic profiling. On one hand, having an adequate amount of high-quality DNA/RNA is essential, and fresh frozen material is the best sample for this purpose, but re-biopsy of patient with advanced disease is not always feasible and it may increase the time to new therapy. Moreover, in patients with metastatic disease, using fresh frozen or FFPE samples might induce a bias due to lesion selection. Liquid biopsy may provide an alternative solution, being non-invasive, and associated with a fast TAT.

Because of the limited sample size, we restricted analyses to univariate comparisons and did not perform multivariable adjustment; observed differences may therefore be influenced by confounding factors such as sex and disease extent (PCI). A significant difference in sex ratio was observed between the Arcagen and AURAGEN cohorts (p = 0.021). This imbalance most likely reflects real-world recruitment patterns and the modest sample size rather than an intrinsic methodological effect, and sex distribution may therefore represent a potential confounder in cross-cohort comparisons [41]We also noticed that, despite careful sample selection, TMB and MSI could not be determined in ∼25% of cases. In this real-world context, the most likely explanations include insufficient tumor cellularity and/or suboptimal nucleic-acid quality/quantity, particularly in FFPE-derived material, which can limit confidence in signature calling; accordingly, some reports may indicate MSI/TMB as “cannot be determined” when the criteria for confident assessment are not met. In our cohorts, TMB was generally low and MSI was overwhelmingly stable; consistent with available data suggesting that MSI-high and high-TMB phenotypes are uncommon in peritoneal mesothelioma, these biomarkers are unlikely to influence routine management for most patients. Nevertheless, MSI-high and/or high TMB remain clinically meaningful in selected cases, as they may support consideration of immune checkpoint inhibitors and can be relevant for trial eligibility or for identifying rare hypermutated phenotypes [6,42,43]

In conclusion, the present study using two different molecular approaches and different types of samples provides a similar proportion of therapeutic targets for PM. The AURAGEN cohort identified a greater number of alterations in genes that currently do not offer access to specific treatment, but require a fresh tumor biopsy, which is not possible for all patients. The Arcagen approach, using already existing FFPE material or liquid biopsy, provided a similar rate of clinically relevant molecular alterations with a faster TAT, allowing treatment adaptation. In summary, our paper highlights the interest of routine molecular profiling for patients with PM as clinically relevant alterations are identified in the majority of tumor samples. For early settings, when surgical samples are easily accessible, a WGS platform such as AURAGEN could be ideal. However, when patient is in therapeutic failure, using a NGS panel on liquid biopsy or the most recent FFPE material may offer in-time therapeutic options for the patient.

## Source of funding

The EORTC SPECTA platform is supported by Alliance Healthcare, which will become Cencora, and F. Hoffman-La Roche Ltd. [Arcagen study (EORTC-1843)]. The AURAGEN platform is supported by FMG2025. The present project is also supported by NetSARC+ INTERSARC+ (INCa), LYriCAN+ (INCa-DGOS-INSERM-ITMO cancer_18003), and EURACAN (EU project 739521).

## CRediT authorship contribution statement

**Seddig Momenkhan:** Writing – review & editing, Writing – original draft, Data curation. **Marie Morfouace:** Writing – review & editing, Writing – original draft. **Vahan Kepenekian:** Writing – review & editing, Investigation. **Olivier Glehen:** Writing – review & editing, Investigation. **Laurent Villeneuve:** Writing – review & editing. **Julio Oliveira:** Resources. **Sabine Tejpar:** Investigation. **Sylvie Isaac:** Data curation. **Juliette Fontaine:** Data curation. **Jean-Yves Blay:** Writing – original draft, Investigation. **Julien Péron:** Writing – review & editing, Data curation, Conceptualization. **Nazim Benzerdjeb:** Writing – review & editing, Writing – original draft, Supervision, Formal analysis, Conceptualization.

## Declaration of competing interest

The authors declare that they have no known competing financial interests or personal relationships that could have appeared to influence the work reported in this paper.
